# Modulation of cell signalling and sulfation in cardiovascular development and disease

**DOI:** 10.1038/s41598-021-01629-0

**Published:** 2021-11-17

**Authors:** Tiago Justo, Antonie Martiniuc, Gurtej K. Dhoot

**Affiliations:** Department of Comparative Biomedical Sciences, The Royal Veterinary College, University of London, Royal College Street, London, NW1 OTU UK

**Keywords:** Biological techniques, Cancer, Cell biology

## Abstract

Sulf1/Sulf2 genes are highly expressed during early fetal cardiovascular development but down-regulated during later stages correlating with a number of cell signalling pathways in a positive or a negative manner. Immunocytochemical analysis confirmed SULF1/SULF2 expression not only in endothelial cell lining of blood vessels but also in the developing cardiomyocytes but not in the adult cardiomyocytes despite persisting at reduced levels in the adult endothelial cells. The levels of both SULFs in adult ischemic human hearts and in murine hearts following coronary occlusion increased in endothelial lining of some regional blood vessels but with little or no detection in the cardiomyocytes. Unlike the normal adult heart, the levels of SULF1 and SULF2 were markedly increased in the adult canine right-atrial haemangiosarcoma correlating with increased TGFβ cell signalling. Cell signalling relationship to ischaemia was further confirmed by in vitro hypoxia of HMec1 endothelial cells demonstrating dynamic changes in not only vegf and its receptors but also sulfotransferases and Sulf1 & Sulf2 levels. In vitro hypoxia of HMec1 cells also confirmed earlier up-regulation of TGFβ cell signalling revealed by Smad2, Smad3, ALK5 and TGFβ1 changes and later down-regulation correlating with Sulf1 but not Sulf2 highlighting Sulf1/Sulf2 differences in endothelial cells under hypoxia.

## Introduction

Development and maintenance of functional myocardium via continuing optimal blood supply is a key requirement for survival^1^. Myocardium undergoes regulated cell proliferation and growth during fetal development and limited hypertrophy during later fetal and postnatal growth to acquire its fully functional pumping capacity. Despite high contractility and relaxation activity throughout life, the postnatal adult cardiomyocytes enter cellular quiescence and down-regulate most growth inducing cell signalling pathways with little scope of postnatal cardiomyocyte repair.

Myocardial infarction is a leading cause of morbidity and mortality^2^ and its mitigation using therapeutic interventions still remains limited requiring improved understanding of key cell signalling events regulating normal cardiovascular development and recovery following myocardial ischemia. The adult myocardium in the injured ischemic border zone following myocardial infarction, however, may be rescued by timely and rapid re-vascularisation of the injured heart^3^. Such re-vascularisation recovery is promoted or inhibited by multiple endogenous cell signalling components implicated in angiogenesis although their precise roles in this process have not been fully defined. Activation of such cell signalling events requires precise regulation and supply of specific angiogenic growth factors such as vegf at right levels as their inappropriate levels can also cause damage by making the surrounding blood vessels permeable. In addition to well characterised receptor tyrosine kinase mediated angiogenic growth factor signalling, the activities of cell signalling molecules are further modulated by the availability and sulfation status of heparan sulfate proteoglycans (HSPGs) that serve as co-receptors for many angiogenic growth factors^4^. While Sulfotransferases initially regulate sulfation characteristics of HSPGs, SULF1 and SULF2 extracellularly edite their sulfation status as they can hydrolyse 6-O sulfates of HSPGs required for cell signalling activities of many angiogenic growth factors. The SULF1 and SULF2 enzymes therefore have the potential to promote or inhibit specific cell signalling activities that are particularly important in cardiovascular development and ischemic injury. Although Sulf1 and Sulf2 genes have been reported to compensate for each other^5,6^, their individual functions in different contexts are not so well characterised. For example, while many studies have reported both SULF1 and SULF2 to be anti-angiogenic^7,8^ some other studies report SULF2 to be pro-angiogenic at least under certain conditions^9^. Their relative expression levels therefore may not only enhance or inhibit cell signalling but also keep their cell signalling activities regulated as required.

In this study, we examined the expression and possible roles of both SULF1 and SULF2 during normal human fetal development of blood vessels and myocardium to explore their positive or negative association with growth of both these tissue types and their relationship to hypoxia or ischemia. SULFs additionally have also been reported to regulate cell signalling pathways implicated in inflammation, a critical component of myocardial injury following infarction^10^. SULF1/SULF2 roles in cardiovascular development appeared quite clear as both these enzymes were highly expressed in developing cardiomyocytes and blood vessels during early growth period but with hardly any detectable levels in the normal adult or injured cardiomyocytes following myocardial infarction. Despite their known anti-angiogenic activities during angiogenesis both SULF1 and SULF2 were found to be co-expressed in endothelial cell lining of fetal blood vessels at high levels but at reduced and yet persistent levels of expression in the endothelial lining of normal adult blood vessels.

The role of SULF1 and SULF2 enzymes in blood vessel growth was further investigated following myocardial infarction and during highly dysregulated haemangiosarcomic growth described as a rare human endothelial cell cancer but a fairly common canine endothelial cell cancer metastasising to many different tissues including right atrium of the adult heart. Despite their known inhibitory roles described in VEGF/FGF cell signalling regulation^8,11^, increased and variable SULF1 and SULF2 expression was observed during active phases of fetal endothelial cell growth, ischemic injury and highly dysregulated haemangiosarcomal growth^12^ with a potential to regulate their angiogenic activities.

## Results

### Sulf1/Sulf2 mRNA expression in developing human fetal cardiovascular tissues

**Figure 1:**
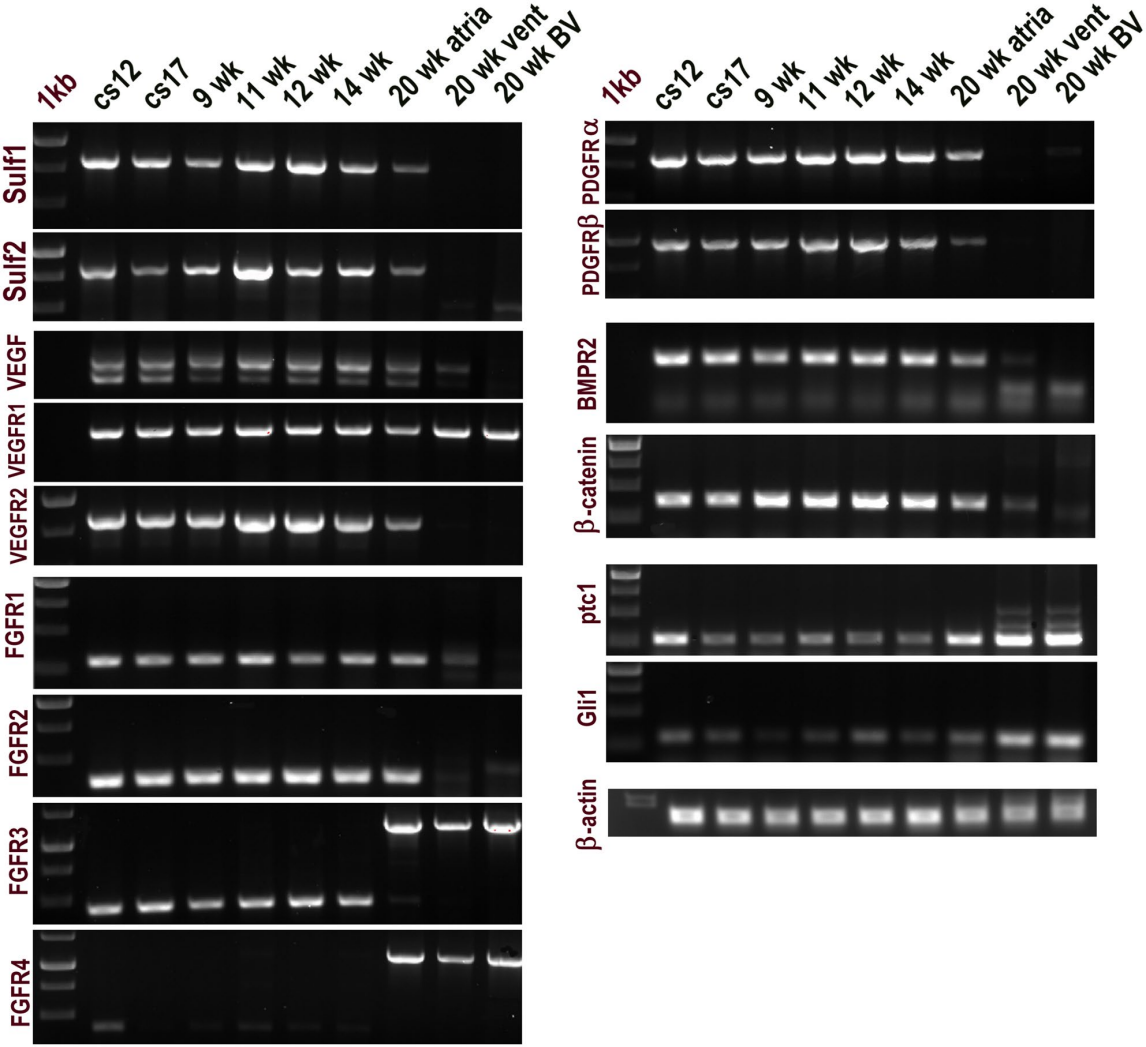
RT PCR analysis showing expression levels of different mRNAs as indicated during different stages of human myocardial development. *BV* blood vessel, *vent* ventricle, *cs* carnegie stage, *wk* week.

Levels of Sulf1 and Sulf2 mRNA expression examined by RT PCR analysis of developing heart seemed generally similar but not identical with Sulf1 showing maximum expression at 12 weeks with Sulf2 maximal expression a week earlier (Fig. 1 and Table 1). Sulf1 and Sulf2 mRNA levels were very low or generally undetectable in 20 week fetal ventricle although low level expression of both Sulfs was still apparent in 20 week fetal atrium indicating later development of atrium when compared with the ventricle. Vegf and vegfr2 as well as pdgfrα, pdgfrβ, bmpr2 & β-catenin expression profiles generally correlated with Sulf1 and Sulf2 expression profiles while vegfr1 was expressed at the same level throughout this developmental period. The expression patterns of different FGF receptor mRNAs in contrast varied considerably during this developmental period. For example, while expression of fgfr1 and fgfr2 was generally similar to Sulfs and many other signalling components in being higher during early cardiovascular developmental phase, fgfr3 and fgfr4 showed the expression of distinct variants in such cardiovascular tissues with high molecular weight fgfr3 and fgfr4 variants being apparent in only older (20 week) atrium, ventricle and blood vessels (Fig. 1, Table 1 and Supplementary Information). The expression of shorter fgfr3 variant in contrast was apparent during cs12—14wk heart development but with shorter variant of fgfr4 variant being apparent in only cs12 cardiac sample. Unlike most receptor tyrosine kinase mediated cell signalling pathways, Hedgehog signalling in contrast appeared to be upregulated during later development as shown by increased ptc1 and gli1 expression (Fig. 1, Table 1).

**Table 1:** This table related to Fig. 1.

Sulf1	69.48	59.36	39.01	69.02	83.18	49.67	20.29	0.43	0
Sulf2	53.05	30.39	50.83	97.59	57.17	57.72	22.49	0	0
VEGF120	43.54	37.61	16.69	22.01	30.12	27.62	21.68	4.01	2.74
VEGF165	46.75	57.74	50.32	70.02	64.93	64.95	36.63	14.1	3.75
VEGFR1	63.16	66.6	64.03	73.61	66.58	62.4	41.86	65.79	76.33
VEGFR2	80.88	75.84	78.09	106.77	103.23	73.02	37.86	0.25	0.48
FGFR1	52.92	45.59	46.91	59.36	37.62	47.09	43.94	20.71	15.45
FGFR2	77.18	80.31	78.92	85.73	91.45	79.04	71.25	11.9	13.71
FGFR3-a	0	0	0	0	0	0	87.58	54.89	83.17
FGFR3-b	47.14	55.95	37.43	48.78	56.31	44.46	0	0	0
FGFR4-a	0	0	0	0	0	0	56.71	34.18	72.77
FGFR4-b	5.66	0	0	0	0	0	0	0	0
PDGFRα	76.93	71.49	69.77	86.09	86.41	73.91	43.21	0	0
PDGFRβ	51.37	40.49	50.48	66.39	70.51	48.12	13.64	0	0
BMPR2	84.61	79.02	62.26	81.1	78.81	79.82	49.02	11.51	3.8
β-catenin	69.13	66.77	86.27	84.92	92.39	82.97	50.83	9.98	0
ptc1	70.16	31.51	27.03	40.3	32.9	31.79	74.57	102.29	107.63
Gli1	23.5	15.41	4.44	11.85	25.06	13.42	28.59	64.66	74.92
β-actin	117.2	101.8	91.26	96.19	102.44	116.67	96.82	90.42	93.96

Levels of different mRNAs during different fetal developmental stages of human cardiovascular tissues.

### SULF1/SULF2 cellular expression in developing human myocardium and blood vessels

**Figure 2:**
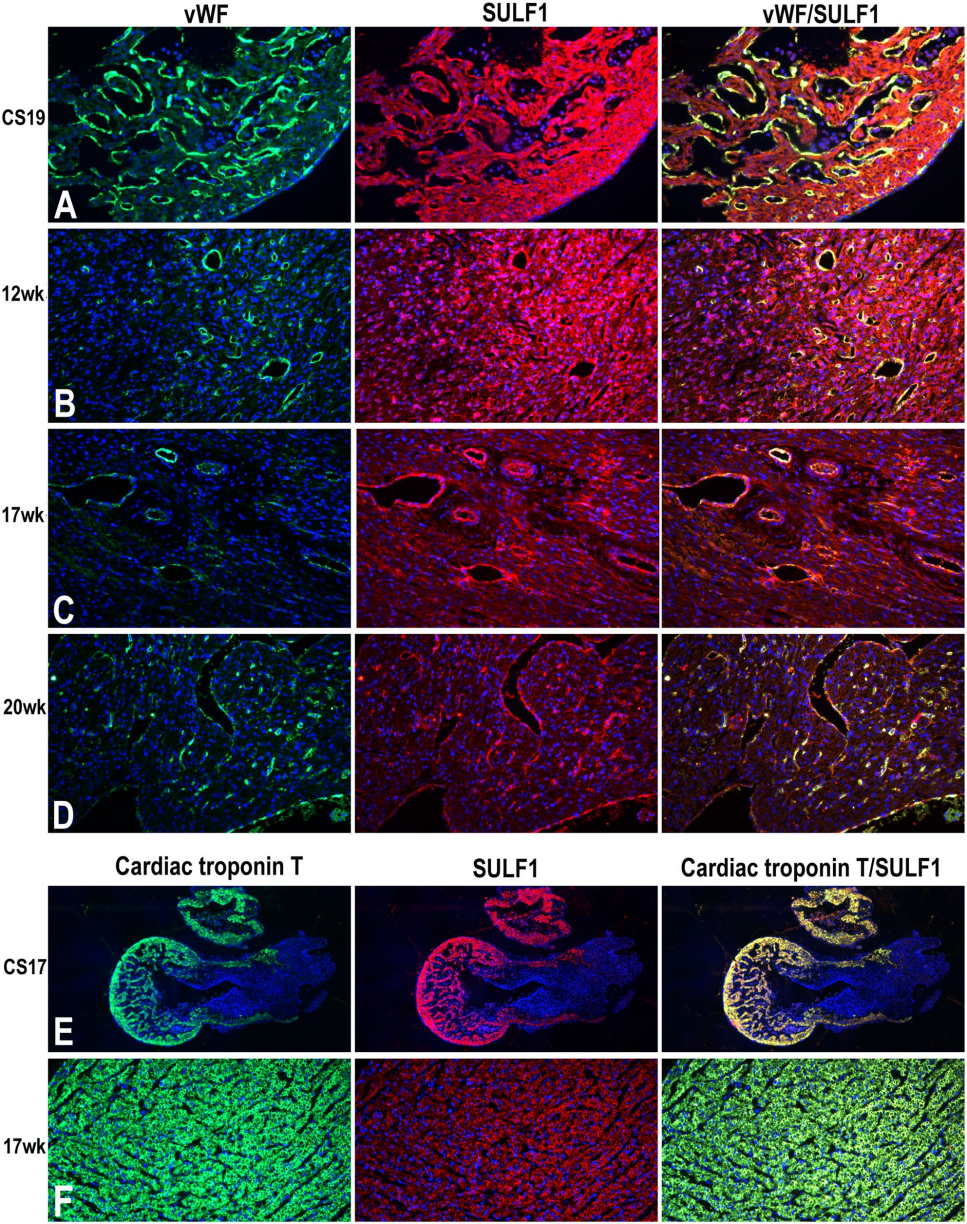
Changing levels of SULF1 and vWF expression detected by double immunofluorescence during cs19, 12 week, 17 week and 20 week of growth highlighting SULF1 and vWF co-expression in endothelial cells **(A–D)**. SULF1 expression unlike vWF is also observed in cardiomyocytes. **(E,F)** demonstrate SULF1 co-expression in cardiac troponin T positive cardiomyocytes.

**Figure 3:**
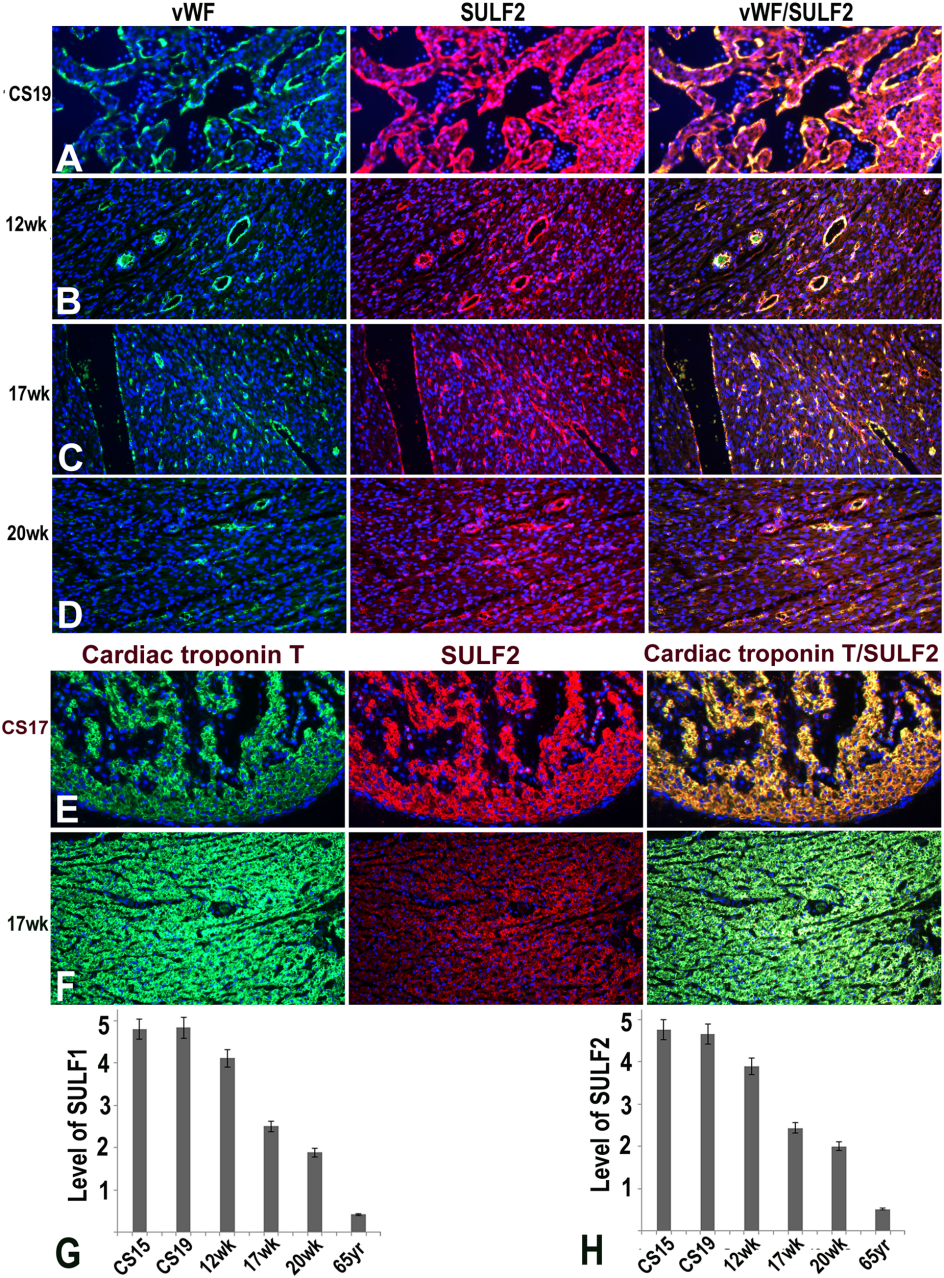
Changing levels of SULF2 and vWF expression detected by double immunofluorescence during cs19, 12 week, 17 week and 20 week of growth highlighting SULF2 and vWF co-expression in endothelial cells **(A–D)**. SULF2 expression unlike vWF is also observed in cardiomyocytes. As was the case for SULF1, **(E,F)** also demonstrate SULF2 co-expression with cardiac troponin T in cardiomyocytes. **(G,H)** demonstrate changing levels of SULF1 and SULF2 expression quantified using volocity software in the adult and embryonic/fetal myocardial development.

SULF1 protein expression was apparent in fetal myocardium and endothelial cell lining of developing blood vessels detected by immunocytochemical staining (Fig. 2). The SULF1 expression in endothelial cell lining was confirmed by its co-expression with vWF immunocytochemical staining although the vWF expression unlike SULF1 was restricted to only endothelial cell lining while SULF1 was also highly expressed in cardiomyocytes. The SULF1 expression in blood vessels was maintained although at reduced levels during later development e.g. at 20 weeks of gestation but the level of SULF1 in myocardium was markedly reduced during later developmental stages as for example at 20 weeks. SULF2 expression in developing myocardium using standard immunocytochemical procedure appeared generally similar to SULF1 expression at these stages as for example is shown for 19cs, 12wk, 17wk and 20wk developing myocardium (Fig. 3). Normal 20 week fetal atrium and fetal ventricle, however, showed quantitative differences indicating later development of atria compared with the ventricle as relative SULF1/SULF2 levels in atria were retained for longer. SULF2 expression in endothelial cell lining was also confirmed by its correlation with vWF immunocytochemical staining (Fig. 3). SULF2 expression as shown for SULF1 (Fig. 2) was not restricted to only blood vessel lining but was also apparent in developing myocardium showing co-expression with cardiac troponin T not only at cs17 embryonic but also at later developmental stage of 17 weeks (Fig. 3) although with reduced SULF levels during later development.

### SULF1/SULF2 ischemic response following human myocardial infarction or murine coronary occlusion

**Figure 4:**
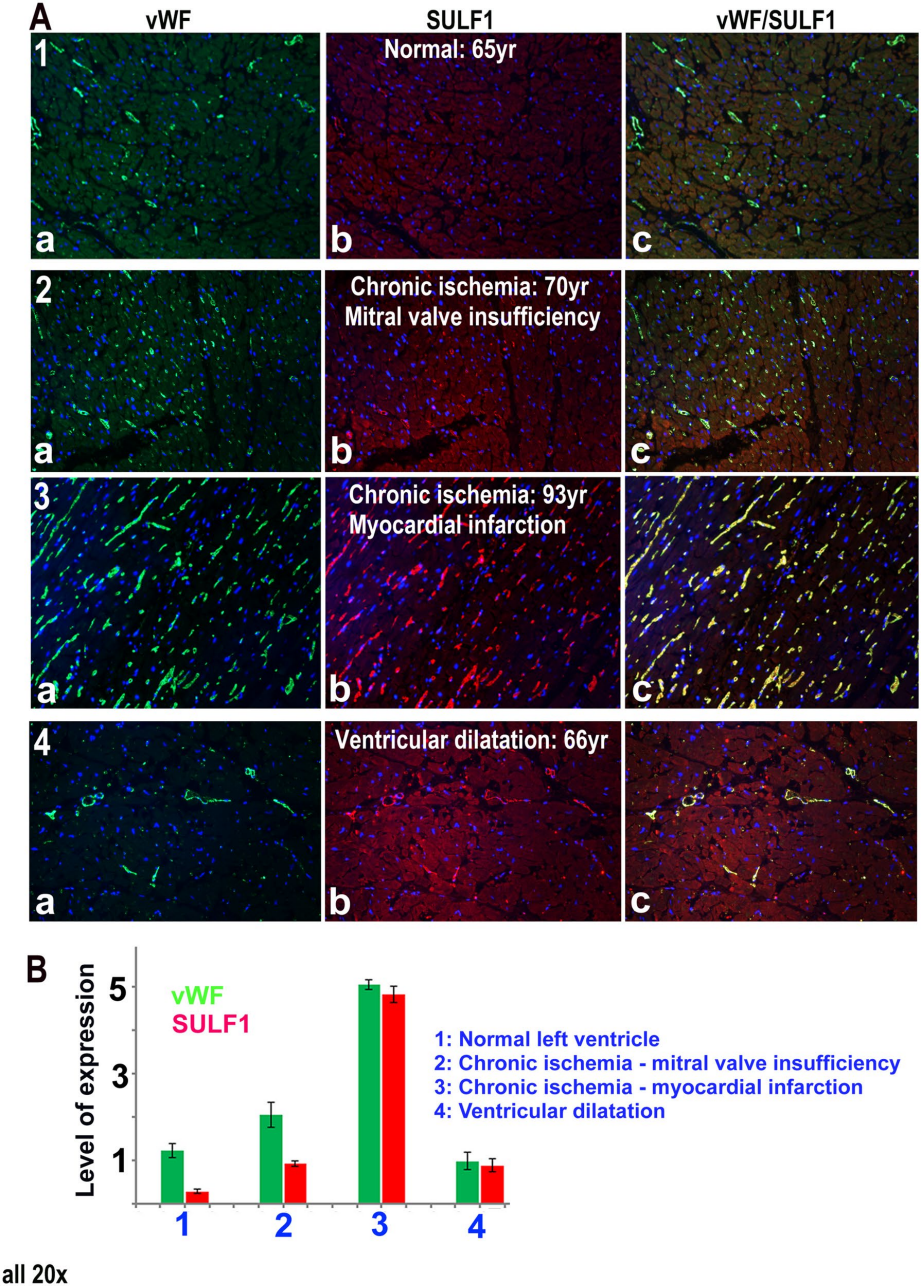
**(A)** Levels of vWF and SULF1determined by double immunofluorescence in normal (1) and ischemic heart due to mitral valve insufficiency (2) or myocardial infarction (3) and during ventricular dilatation (4). **(B)** Quantification of vWF and SULF1 expression levels using volocity software.

**Figure 5:**
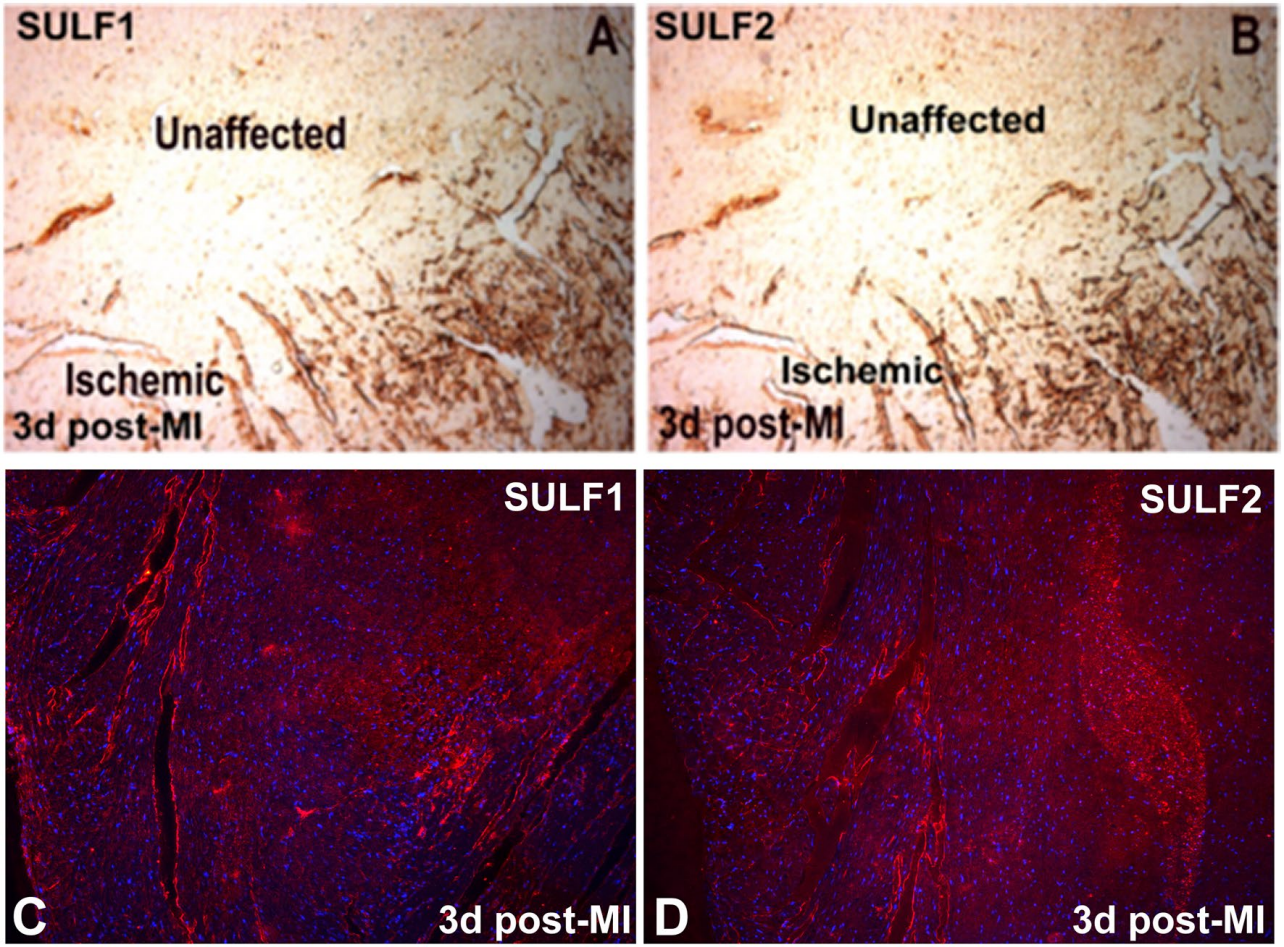
Ventricular expression pattern of SULF1 and SULF2 using immunoperoxidase **(A,B)** and immunofluorescence **(C,D)** procedure following coronary occlusion for 3 days **(A–D)**. Both immunoperoxidase and immunofluorescence procedures demonstrate only restricted regional pattern of SULF1 and SULF2 expression with no or barely detectable levels in distant regions.

While SULF1 and SULF2 expression was undetectable in the normal adult cardiomyocytes using immunocytochemical staining, a low level expression of both these enzymes was detectable in the endothelial lining of some adult blood vessels. SULF1 (Fig. 4 (1a)) and SULF2 (not shown) staining of blood vessels in the normal adult heart, however, was considerably lower than the vWF (Fig. 4(1b)) levels using similar immunocytochemical procedure indicating marked reduction in the levels of both SULF enzymes compared with earlier fetal stages of development. The levels of SULF1/SULF2, however, showed marked variation in blood vessel staining of human cardiac samples of different patients with cardiovascular problems as shown for SULF1 in Fig. 4. Many myocardial samples generally showed increased SULF1/SULF2 expression in ischemic hearts following single or multiple myocardial infarctions irrespective of their age. This was further apparent from increased but only regional SULF1/SULF2 expression in murine blood vessels following coronary occlusion (Fig. 5). Despite increased SULF1/SULF2 expression in some blood vessels, upregulation of these enzymes was not apparent in the cardiomyocytes. Only rarely a trace amount of SULF1/SULF2 was detectable on the cell membrane of a few myocardial cells in some patients (not shown).

### Marked TGFβ and SULF1/SULF2 up-regulation in atrial haemangiosarcomal endothelial cells

**Figure 6:**
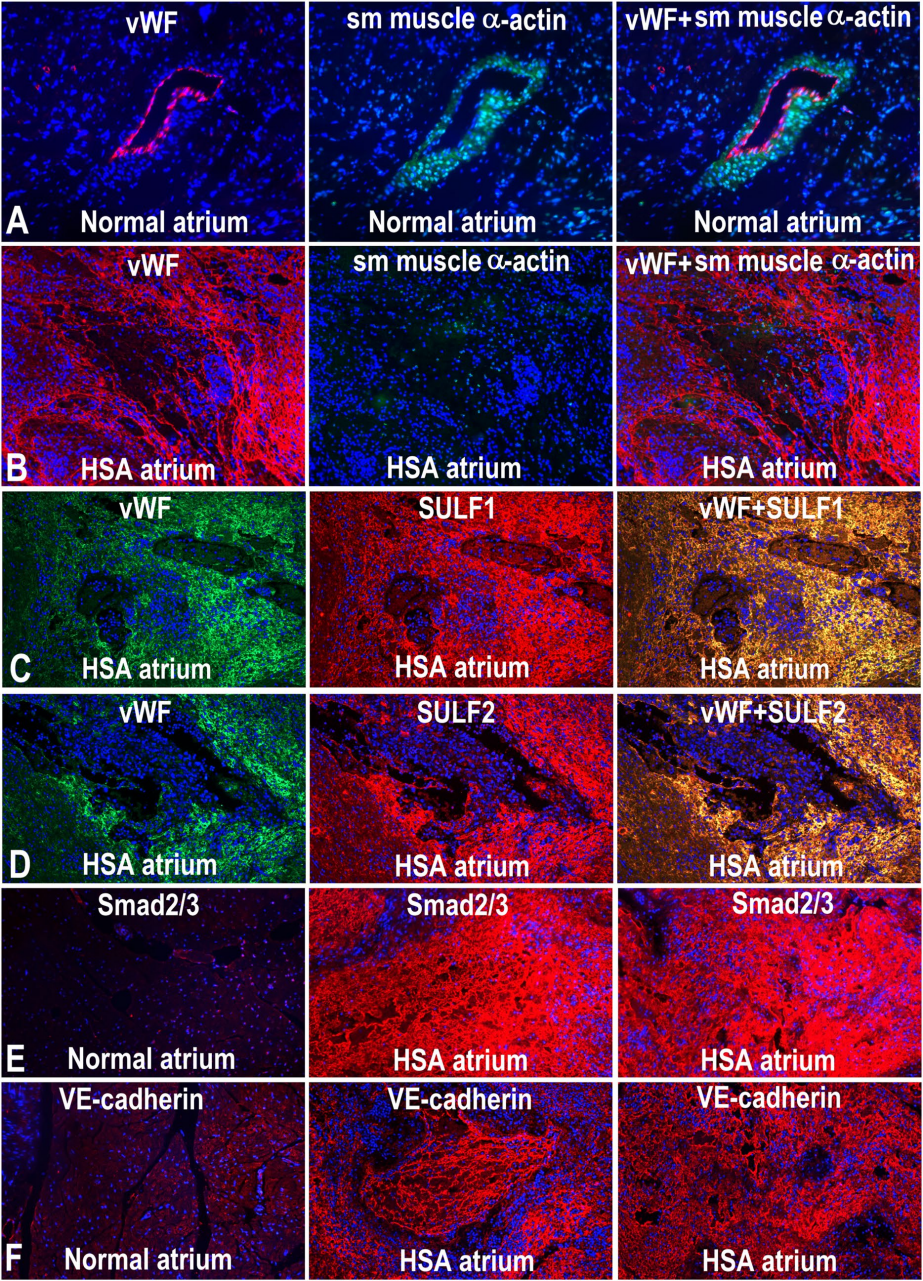
Pattern of vWF and smooth muscle α-actin in a blood vessel of normal unaffected **(A)** and haemangiosarcomic right atrium **(B)** determined by double immunofluorescence procedure. Double immunofluorescence also demonstrates co-expression of both SULF1 **(C)** and SULF2 **(D)** with vWF as well as markedly increased expression of Smad2/3 in haemangiosarcomal atria (HSA) unlike the control atrium **(E)** as is also the case for markedly increased VE-cadherin in HSA **(F)** in such atria.

As is the case with most haemangiosarcoma cells metastasising to different tissue types, right atrial haemangiosarcoma was characterised by abundance of small and large dysfunctional clusters of abnormal endothelial tubes identified from vWF and VE-cadherin co-expression but lacking smooth muscle cell recruitment (Fig. 6). Many such blood vessel clusters also included collections of blood cells, mainly erythrocytes that showed little resemblance to normal blood vessels due to virtual absence of mural cell recruitment (Fig. 6) and fragility of such structures characterising regional haemorrhages. High levels of Smad2/3 activation in such dysfunctional blood vessels compared with normal blood vessels indicated increased TGFβ cell signalling in such endothelial cell tubes (Fig. 6).

### Sulf1/Sulf2 and cell signalling responses of HMec1 endothelial cells to in vitro hypoxia

**Figure 7:**
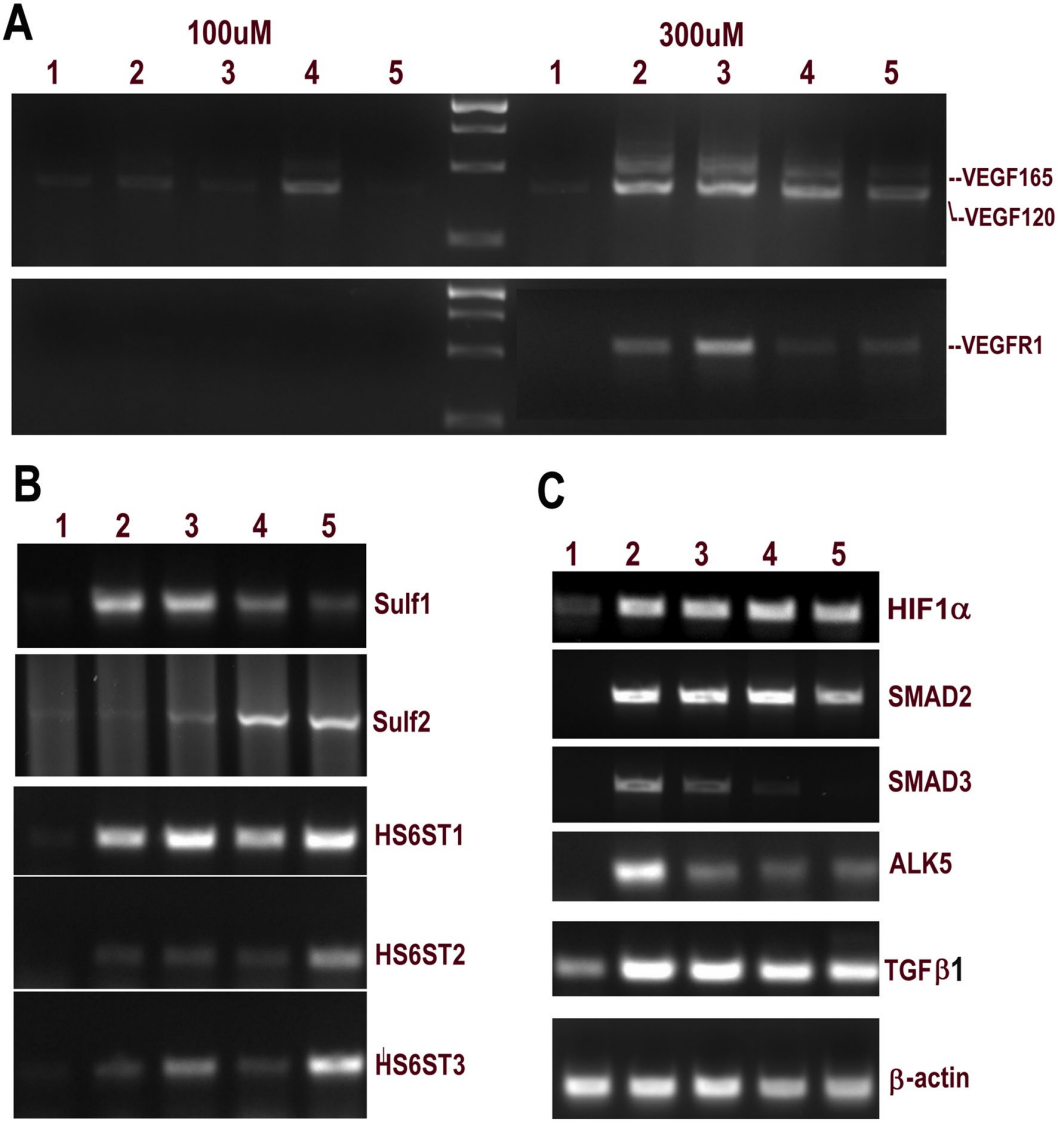
RT PCR analysis showing changes in vegfr1 and vegf variants in HMec1 endothelial cells at lower (100 µM) and higher (300 µM) levels of hypoxia in untreated (1) and CoCl2-treated cells for 3 h (2), 24 h (3), 72 h (4) and 96 h (5) of hypoxia. Changes in Sulf1, Sulf2, sulfotransferases and components of TGFβ cell signalling and β-actin were examined following treatment with 300 µM CoCl2. CoCl2 induced hypoxia in such cells was confirmed by rapid activation of HIF1α.

To examine if some changes in cell signalling or Sulf1/Sulf2 levels during in vivo ischemia were also apparent during in vitro hypoxia, we exposed HMec1 human endothelial cells to 100 µM and 300 µM CoCl2 chemical hypoxia for different lengths of time to examine changes in VEGF levels. CoCl2 induced hypoxia was confirmed by rapid HIF1α activation (Fig. 7, Table 2 and Supplementary Information). The control HMec1 cells only express a trace amount of the shorter VEGF_120_ isoform that at 100 µM only increased a little at 3 days without any up-regulation of VEGF_165_ isoform, the most potent isoform, or recruitment of VEGFR1 unlike rapid changes observed in both VEGF_120_ and VEGF_165_ isoforms and recruitment of VEGFR1 at 300 µM CoCl2 (Fig. 7 and Table 2). Sulf1/Sulf2 changes also became apparent under such conditions as Sulf1 levels rapidly increased upon hypoxia that later dropped down to low levels. Sulf2, in contrast, however, showed delayed and only gradual up-regulation of Sulf2 throughout this hypoxic time scale investigated in this study (Fig. 7). In addition to Sulfs, we also observed the hypoxic modulation of heparan sulfate 6-O sulfotransferases (HS6ST-1, -2, and -3) as levels of all three sulfotransferases increased during prolonged hypoxia although HS6ST-1 showed much higher increases at all stages. Such changes in sulfation under hypoxia led to rapid and marked upregulation of TGFβ cell signalling detected by high Smad2/Smad3, ALK5 and TGFβ mRNA levels (Fig. 7 and Table 2).

**Table 2:** This table related to Fig. 7.

CoCl2	mRNA	Time under hypoxia using CoCl2				
		0 h	3 h	24 h	72 h	96 h
100 μM	VEGF120	0.98	7.12	1.6	22.21	2.25
	VEGF165	0	0	0	0	0
	VEGFR1	0	0	0	0	0
300 μM	VEGF120	10.63	65.45	75.72	59.57	26.58
	VEGF165	2.71	42.82	49.26	29.62	5.7
	VEGFR1	5.15	30.29	58.72	11.58	17.04
	Sulf1	13.99	87.99	88.27	46.72	24.82
	Sulf2	5.69	4.35	17.36	58.89	60.1
	HS6ST1	0	82.01	117.86	81.75	123.42
	HS6ST2	0	12.14	17.8	15.6	61.32
	HS6ST3	2.8	15.34	45.97	17.82	107.29
	HIF1α	25.87	88.92	90.02	98.68	87.04
	Smad2	0	94.08	97.17	101.47	70.19
	Smad3	0	43.1	19.58	3.06	0
	ALK5	0	119.27	73.08	29.07	45.7
	TGFβ1	47.13	135.16	128.06	114.47	116.19
	β-actin	112.02	111.12	112.09	82.11	88.61

Levels of different mRNAs in HMec1 cells at different time points following 100 μM or 300 μM CoCl2 treatment.

## Discussion

Both RT PCR and immunocytochemical analyses detected high levels of SULF1/SULF2 expression during early fetal development but gradual down-regulation during later fetal and postnatal stages to virtually undetectable levels in the adult cardiomyocytes indicating high SULF1/SULF2 activity relating to early myocardial cell proliferation enhancing or regulating related cell signalling pathways^13^ but not hypertrophy or maintenance of quiescent cell phenotype. Such expression profile is similar to high SULF1/SULF2 expression reported in normal fetal skeletal muscle but undetectable levels in normal adult muscle and yet marked up-regulation during injury-induced skeletal muscle regeneration^14^. The SULF1/SULF2 re-activation during adult skeletal muscle injury represents a marked difference between adult skeletal muscle versus myocardium highlighting lack of cardiac stem cell repair of injured cardiomyocytes^14^. Neither the normal adult skeletal muscle fibres or quiescent myogenic stem cells (satellite cells) express any SULF1 or SULF2. SULF1/SULF2 re-activation in striated muscles therefore appears to relate to muscle progenitor/stem cell growth apparent in injured skeletal muscle but not in adult myocardium. SULF1/SULF2 enzymes thus are not up-regulated in postnatal cardiomyocytes following down-regulation during later fetal development.

Reduction in SULF1/SULF2 expression levels in the adult endothelial lining of blood vessels in comparison with fetal development indicates reduced endothelial cell activity and related cell signalling activities although low level persistence could enable rapid up-regulation upon ischemic injury. Persistence of low level SULF expression in adult endothelial cells could also inhibit cell proliferation to maintain endothelial cell differentiation and thus cell quiesence. High endothelial SULF1/SULF2 levels during early fetal growth could relate to high levels of regulated cell signalling controlling cell proliferation while low level persistence in adult endothelium represents not quiescent but being amenable to rapid cell signalling up-regulation upon ischemia. This could thus relate to increased SULF1/SULF2 expression during ischemia^15^ to regulate increased angiogenic growth factor cell signalling or indeed to regulate the activities of key receptor tyrosine kinase cell signalling pathways known to be inhibited by SULFs. For example, Narita et al. (2006) have demonstrated the inhibition of both VEGF and FGF signalling by SULF1 and SULF2 as these enzymes remove 6-O sulfates of HSPGs required for their cell signalling^8^. Uchimura et al. (2006) have also shown Sulf-2 to block VEGF, FGF-1, and SDF-1 binding to heparin and thus inhibit their cell signalling activities^16^. Vegf is one of a key endothelial cell mitogen known to regulate blood vessel development and homeostasis that interacts with one or more vegf receptor tyrosine kinases (vegfr1, vegfr2 and vegfr3) and with HSPG and neuropilin co-receptors^17^. RT PCR analysis of developing myocardium showed closer correlation of Sulf1/Sulf2 expression with vegfr2 than vegfr1 during human fetal development although vegf cell signalling has been described to be inhibited by both SULF1 and SULF2^8,11,16^. This differs from vegf receptor change upon endothelial cell hypoxia showing correlation with vegfr1 instead of vegfr2. High SULF1 and SULF2 levels during fetal development thus may not correlate with their pro-angiogenic but rather regulatory inhibitory roles to control receptor tyrosine kinase cell signalling to limit cell proliferation. It is, however, still unclear whether SULF1 and SULF2 have identical or different roles during angiogenesis as these enzymes have been reported to compensate for each other’s activities^5^.

Myocardial infarction due to blockage of a coronary artery depriving oxygen leads to myocardial ischemia. Mild Ischemia could promote angiogenic cell signalling to restore angiogenic recovery while high level ischemia induces myocardial necrosis without beneficial angiogenesis. Inflammation also plays an important role in the pathogenesis of myocardial infarction that itself is regulated by multiple cell signalling pathways including their modulation by SULF1/SULF2 enzymes^10^. Human myocardial infarctions in this study showed variable levels of SULF1/SULF2 increases in different patients or different parts of the same heart that could relate to the length of ischemia and distance from the ischemic injury. Experimental murine heart coronary occlusion also confirmed increased angiogenesis in only regions adjacent to myocardial ischemia. Ischemia, nevertheless promoted SULF1/SULF2 expression with a potential to regulate angiogenesis. A recent study also reported the upregulation of both SULFs following experimental murine coronary artery ligation although with varying cellular expression patterns e.g. Sulf2 expression was reported in monocytes and macrophages but with Sulf1 expression being restricted to endothelial cells and fibroblasts^10^. Increased SULF1/SULF2 expression in ischemic myocardium thus could regulate angiogenesis even though most of the receptor tyrosine kinase signalling pathways promoting angiogenesis are known to be inhibited by SULFs^8,11,16^. The effects of SULF1 and SULF2 on TGFβ cell signalling, however, are less clear showing both positive and negative effects demonstrating enhancement as well as inhibitory activities^11,18,19^.

Unlike normal adult endothelial cells expressing only a trace amount of SULFs, SULF1/SULF2 expression was markedly up-regulated in canine endothelial cell cancer, described as right atrial haemangiosarcoma. Simultaneous increase in TGFβ cell signalling detected by Smad2/Smad3 activation in such cells could be responsible for SULF1/SULF2 enhanced TGFβ cell signalling promoting haemangiosarcomal cell growth although this needs to be further examined using in vitro gain and loss of function analyses. Hemangiosarcoma of the heart that is quite common in dogs but rare in human is known to affect parts of the right atrium in which fragile blood vessels grow abnormally large^20–22^. The absence of mural cell recruitment despite increased but unregulated excessive endothelial cell growth highlights the dysregulated cell signalling and associated haemorrhages in such cancers resulting from dysregulated TGFβ/VEGF cell signalling. A further molecular study of different stages of larger number of atrial haemangiosarcoma samples may reveal the role of some SULF1/SULF2 complexity under such conditions. The co-expression of both SULF1 and SULF2 in vWF-positive cells in vivo endothelial cells during normal fetal development and in vitro endothelial cell hypoxia indicates the involvement of these enzymes in the regulation of blood vessel growth, repair and maintenance.

Hypoxia occurs not only under pathological conditions but is also observed during normal embryonic development^23^. Early fetal hypoxia has been reported to lead to growth restriction and myocardial thinning^24^. In vitro hypoxia of endothelial cells in this study clearly demonstrated the modulation of both Sulf1/Sulf2 and some growth factors that may help explain in vivo changes observed upon myocardial infarction or disease. Dynamic quantitative and qualitative changes in the levels and different variants of VEGF upon in vitro hypoxia also highlight the transient and regional cell signalling activation under such conditions. Hypoxic modulation was not limited to only VEGF cell signalling but also Sulf1/Sulf2 and TGFβ cell signalling highlighted by Smad2/Smad3 activation in such cells. Angiogenesis therefore may be regulated not only by vegf but also by SULFs and TGFβ cell signalling representing different steps of the related signalling cascade and thus possible additional therapeutic targets.

## Materials and methods

### Human cell, fetal and myocardial infarction tissue samples

Fetal tissues fixed in 4% paraformaldehyde or paraffin embedded human fetal tissues for sectioning were provided by HDBR as were the fresh frozen cardiovascular samples for RNA analyses. Some human cardiac paraffin embedded tissue array sections for antibody stains were purchased from US Biomax and BioCat. The paraffin embedded normal and hamangiosarcomic canine cardiac tissue sections were provided by the RVC diagnostic laboratory. Mouse hearts following coronary occlusion were kindly provided by Professor Ajay Shah’s group^25,26^. HMec1 human endothelial cell line for hypoxic exposure was obtained from ATCC and cultured as recommended by supplier instructions.

### Immunocytochemistry

The specificities of both SULF1 and SULF2 peptide antibodies to their non-aligning sequences have previously been described in our earlier publications establishing no cross-reactivity between SULF1 and SULF2^27–33^. For example, SULF1 and SULF2 antibody stained immunoblots of membrane, nuclear and cytosolic fractions of neuronal cell types showed some similarities but also marked qualitative and quantitative differences in SULF1 and SULF2 staining as is shown by detection of cytosolic SULF2 but not SULF1 in same N2A cell fractions^30^.Three sets of all paraffin tissue sections were used for antibody stain that were pre-treated with permeabalisation buffer for 15 min at room temperature followed by incubation with 10% foetal calf serum (FCS) for 30 min before treatment with different primary antibodies at different dilutions e.g. SULF1 (1/200), SULF2 (1/100), vWF (1/200), cardiac troponin I (1/100), and cardiac troponin T (1/100), rabbit anti VE-cadherin (1/100). The specificities of the SULF1 antibody C and SULF2 antibody D have previously been described^27–33^. Rabbit anti-Von Willebrand Factor antibody and cardiac troponin I and cardiac troponin T antibodies were purchased from Abcam while rabbit anti VE-cadherin and rabbit ant smad2/3 were purchased from Cell signalling. The binding of all rabbit primary antibodies to SULF1, SULF2, vWF, cardiac troponin T, VE-cadherin and Smad2/3 was detected using streptavidin Alexa Fluor 594 or Alexa Fluor 488 fluorochrome bound to biotin-linked goat anti-rabbit immunoglobulins as previously described^31^. The binding of sheep anti cardiac troponin I was detected using streptavidin Alexa Fluor 488 fluorochrome bound to biotin-linked goat anti sheep immunoglobulins as previously described^31^. Sections treated with pre-immune rabbit sera were similarly incubated with fluorochrome-labelled secondary antibodies as controls (not shown). All primary antibody incubations overnight at 4 °C were followed by secondary antibody incubations for 1 h each at room temperature. Following four PBS washes between and after each incubation, labelled tissue sections were mounted in polyvinyl alcohol mounting medium with DABCO and 2.5 μg/ml DAPI for nuclear visualisation to photograph images using a Leica DM4000B fluorescent microscope. Immunoperoxidase procedure was also used to stain for SULF1 and SULF2 expression following endogenous peroxidase blocking activity by 3% H2O2 for 30 min before incubation with primary antibodies as described previously^32,33^. Tissue sections for right atrial haemangiosarcoma represented four different canine hearts. Level of immunofluorescent stain for different sections was quantified using Volocity software^33^ and statistical analysis was preformed using ANOVA, where data depicting a P value < 0.05 were considered statistically significant.

### RT PCR

RNA from human fetal cardiovascular tissues representing three sets of each developmental stage except cs12 stored at −80 °C was prepared using Trizol (Invitrogen) according to the manufacturer’s instructions. RNA integrity was assessed by electrophoresis using Syber safe staining and OD260/OD280nm absorption ratio (> 1.95). Total RNA (1 μg) for each sample was reverse-transcribed into cDNA with SuperScript II reverse transcriptase (Invitrogen) using random primers (Invitrogen) for RT-PCR analysis with 40 PCR amplification cycles using the following primers: Sulf1: 5′-CGAGGTTCAGAGGACGGATA-3′ & 5′-GCCTCTCCACAGAATCATCC-3′; Sulf2: 5′-CAACTGTGTTCTCCCTGCTGGGT-3′ & 5′- CTGGAGCATGTTGGTGAATTCC -3′; vegf: 5′- GAAGTGGTGAAGTTCATGGATGTC-3′ & 5′-CGATCGTTCTGTATCAGTCTTTCC-3′; vegfr1: 5′-CTGAGAACAACGTGGTGAAGATT-3′ & 5′-CTGACATCATCAGAGCTTCCTGA-3′; vegfr2: 5′-CAACAAAGTCGGGAGAGGAG-3′ & 5′-ATGACGATGGACAAGTAGCC-3′; fgfr1: 5′-TACCACCGACAAAGAGATGG-3′ & 5′-CTGGCTGTGGAAGTCACTCT-3′; fgfr2: 5′-TGGAGCGATCGCCTCACCG-3′ & 5′-CTTCCAGGCGCTGGCAGAACTGT-3′; fgfr3: 5′-CACCACCGACAAGGAGCTA-3′ & 5′-GCTCGAGCTCGGAGACATT-3′; fgfr4: 5′-GGGTCCTGCTGAGTGTGC-3′ & 5′-GGGGTAACTGTGCCTATTCG-3′; pigf: 5′-AAG TG CCG GTC ATG AGG C-3′ & 5′-CTGCATGGTGACATTGGC-3′; pdgfrα: 5'-GGCAGTACCCCATGTCTGAA-3′ & 5'-AGCTTCCAACTGGCTGAAGG-3'; pdgfrβ: 5'-GGCCAGAGCTTGTCCTCAAT-3′ & 5'-AGGGTGCGGTTGTCTTTGAA-3′; bmpr2: 5′-AAAGCCCAGAAGAGCACAGA-3′ & 5′-AGCGATTCAGTGGAGATGAC-3′; β-catenin: 5′- TTCCGAATGTCTGAGGACAAG-3′ & 5′- GTATCAAACCAGGCCAGCTG-3′; gli1: 5′-TGCCTTGTACCCTCCTCCCGAA-3′ & 5′-GCGATCTGTGATGGATGAGATTCCC-3′; ptch1: 5′-TTCTCACAACCCTCGGAACCCA-3′ & 5′-CTGCAGCTCAATGACTTCCACCTTC-3′; Smad2: 5′-AACAGAACTTCCGCCTCTGG-3′ & 5′-ACCGTCTGCCTTCGGTATTC-3′; Smad3: 5′-GGAGACACATCGGAAGAGGC-3′ & 5′-CCCTCCCCATCCCAAGTCTA-3′; ALK5: 5′‐GGGGCGACGGCGTTACAGTGTTTCTGCCAC‐3′ & 5′‐TGAGATGCAGACGAAGCACACTGGTCCAGC‐3′; TGFβ1:5′-AAGTGGATCCACGAGCCCAA-3′ & 5′-GCTGCACTTGCAGGAGCGCAC-3′; HS6ST1: 5′-CATCACCCTGCTACGAGACC-3′ &5′-AAGGGCCGGATGAACTTGAG-3′; HS6ST2: 5′- CCAAGTCAAATCTGAAGCACA-3′ & 5′-CTGGAAATGGGTCTGAAGGA-3′; HS6ST3: 5′- CTTGCGGGAGTTTATGGATTG-3′ & 5′- GGTGCTCTAGCTGCTTGGTGT-3′; HIF1α:5′-CGTTCCTTCGATCAGTTGTC-3′ & 5′-TCAGTGGTGGCAGTGGTAGT-3′; β-actin: 5′-GGCCCAGAGCAAGAGGCATCC-3′ & 5′-AGCAACGTACATGGCTGG -3′. RT PCR products were examined by 2% agarose gel separation. Splice variants for fgfr3 and fgfr4 were cut out and purified to verify their identity by DNA sequencing (GATC Biotech). All RT PCR reactions were repeated 3 times. All RT PCR experiments were repeated at least 3 times but only 1 set of RT PCR gels was used to semi-quantify for representation in Tables 1 and 2 (Supplementary Information).

## Supplementary Information

Supplementary Legends.

Supplementary Figure S1.

Supplementary Figure S2.
