# Supplementary material for: Modulation of cell signalling and sulfation in cardiovascular development and disease
Source: Sci Rep. 2021 Nov 17;11:22424. doi: 10.1038/s41598-021-01629-0 (PMC8599478; doi:10.1038/s41598-021-01629-0)
Supplement: Supplementary file 1 — Supplementary Legends. [file 41598_2021_1629_MOESM1_ESM.docx]

**FIGURE LEGENDS** for Supplementary Figures 1 and Figure 7 showing full length RT PCR agarose gels:

**Supplementary Figure 1**: RT PCR analysis of gene expression (as indicated on each gel) in 9 human fetal cardiovascular samples representing different stages of development. Sample 1=cs12 embryonic heart, 2=cs17 embryonic heart, 3=9wk fetal heart, 4=11wk fetal heart, 5=12wk fetal heart, 6=14wk fetal heart, 7=20wk fetal atria, 8=20wk fetal ventricle, 9=20wk fetal blood vessels.

**Supplementary Figure 2 representing Figure 7**: Effect of hypoxia on VEGF and related genes in HMec1 microvascular endothelial cells analysed using RT PCR analysis. VEGF and VEGFR1 expression is analysed at both 100µM and 300µM concentration of CoCl2 and confirmed by the activation of HIF1α while all other genes as indicated on the gels including Sulf1, Sulf2, sulfotransferases and some TGFβ signalling components were analysed at 300µM concentration of CoCl2. Sample 1 in each case is control untreated cells, sample 2-5 represent different time points following CoCl2 treatment e.g sample 2 = 3hr, 3= 24hr, 4=72hr, 5=96hr.
