# Supplementary material for: Modulation of cell signalling and sulfation in cardiovascular development and disease
Source: Sci Rep. 2021 Nov 17;11:22424. doi: 10.1038/s41598-021-01629-0 (PMC8599478; doi:10.1038/s41598-021-01629-0)
Supplement: Supplementary file 2 — Supplementary Figure S1. [file 41598_2021_1629_MOESM2_ESM.docx]

Supplementary full length gels for Figure 1:
