# Supplementary material for: Modulation of cell signalling and sulfation in cardiovascular development and disease
Source: Sci Rep. 2021 Nov 17;11:22424. doi: 10.1038/s41598-021-01629-0 (PMC8599478; doi:10.1038/s41598-021-01629-0)
Supplement: Supplementary file 3 — Supplementary Figure S2. [file 41598_2021_1629_MOESM3_ESM.docx]

Figure 7. A: Lanes 1 and 2


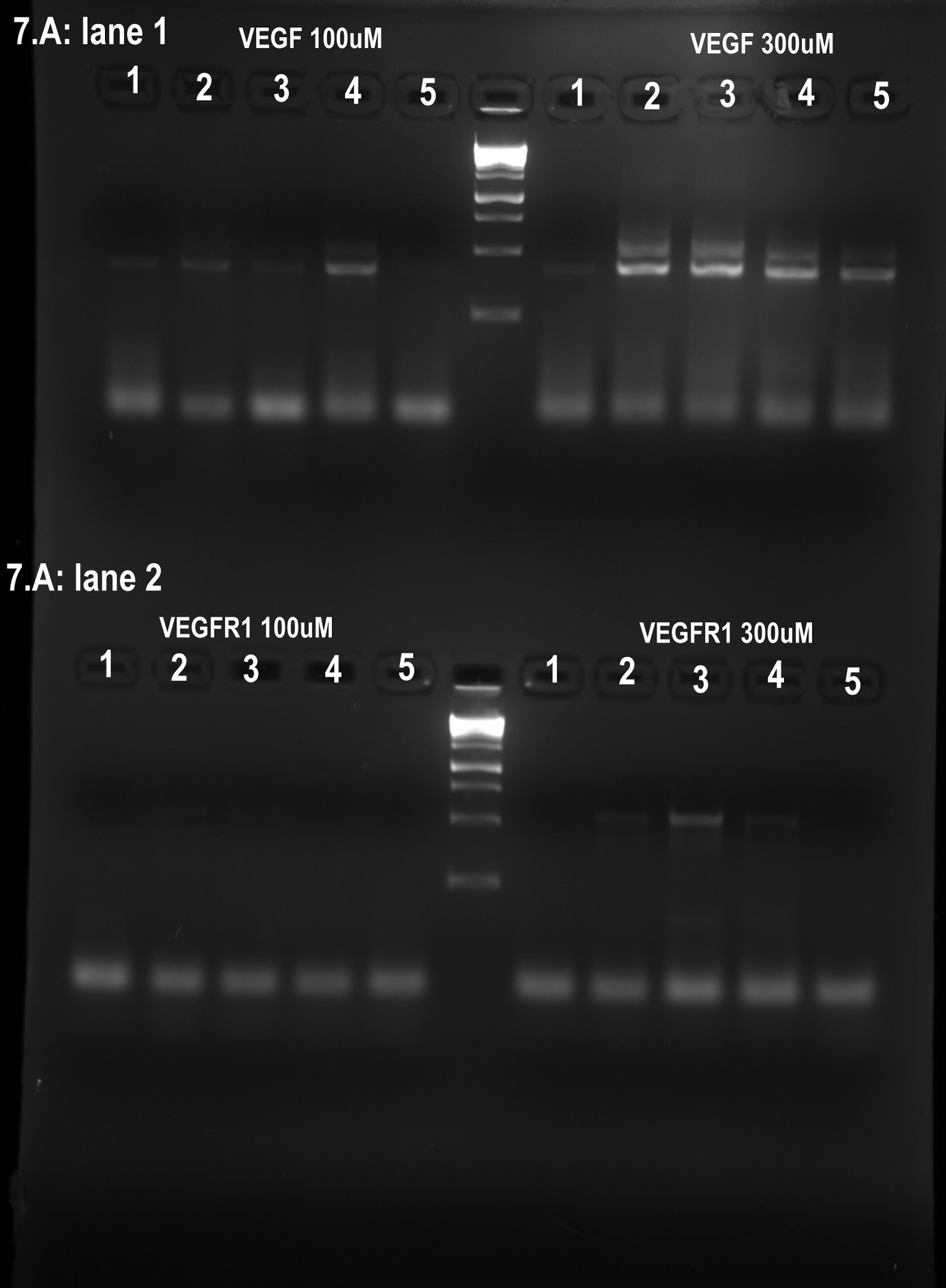


7.B: Lane 1 Sulf1

7.B. Lane 3: **HS6ST1**

7.B: Lane 4 7.B: Lane 5

**HS6ST2 HS6ST3**

**7.C: Lane 1 HIF1α**

**7.C: Lane 2 7.C: Lane 3**

**7.C: Lane 4** **ALK5**

**7.C: Lane 5**

**7.C: Lane 6**
